# Structural Bioinformatics: exciting times in a rapidly evolving field

**DOI:** 10.1002/2211-5463.13968

**Published:** 2025-02-03

**Authors:** Cláudio M. Soares, Diana Lousa

**Affiliations:** ^1^ Instituto de Tecnologia Química e Biológica Universidade Nova de Lisboa Oeiras Portugal

## Abstract

Structural Bioinformatics is a multidisciplinary field at the intersection of chemistry, physics, biology, and computer science. Its methods and approaches have been crucial in advancing scientific knowledge and breaking new ground. By extracting valuable insights from experimental data, generating predictions that accelerate research and reduce costs, and offering novel insights and testable hypotheses, Structural Bioinformatics plays a pivotal role in modern science. The field is undergoing transformative advancements, driven by developments in areas such as Artificial Intelligence. This special issue of *FEBS Open Bio* features five mini‐review articles by leading experts, providing an overview of some of the most recent advancements in Structural Bioinformatics.

**Abbreviations**
AI: artificial intelligence
MD: molecular dynamics

The award of the 2024 Nobel Prize in Chemistry to David Baker, Dennis Hassabis, and John Jumper for their pioneering work in protein design and structure prediction underscores the transformative advancements in the field of Structural Bioinformatics [1, 2].

This field, which crosses borders between chemistry, physics, biology, and computer science, was born in the second half of the 20th century when researchers began using computers to refine protein structures determined by X‐ray crystallography [3]. The field evolved at a fast pace since its inception and branched into a vast array of subtopics, which use different computational methodologies and approaches to address key questions related with structural properties of biological molecules. Some of the most relevant examples include protein structure and function prediction, analysis of protein–protein and protein–ligand interactions, study of protein dynamics and conformational diversity, and protein design and engineering. Recent developments in Artificial Intelligence (AI) are having a major impact in this field and have fueled the development of a large number of new methods that are either purely based on AI or combine these methods with approaches based on homology data (e.g., AlphaFold) or with physics‐based approaches (e.g., Molecular Dynamics [MD] simulations with AI‐based force fields). We are living in exciting times in the world of Structural Bioinformatics, and these are also times when we should take stock of the situation, reflect on the current achievements and challenges, and forecast the future directions. In this context, we are very pleased to introduce this special “In the Limelight” issue of *FEBS Open Bio*, which contains multiple articles featuring the different facets of the rapidly expanding field of Structural Bioinformatics.

In a very timely mini‐review article entitled “An outlook on structural biology after AlphaFold: tools, limits and perspectives,” Rosignoli *et al*. [4] share their perspective into the impact that AlphaFold is having on the current landscape of Structural Bioinformatics and discuss how the field is adapting to this revolution. An interesting section is devoted to the growing need of making models such as AlphaFold accessible not only to the scientific community but also to increase the interpretability of the models and devise solutions that “provide transparent insights into the decision‐making processes of these models, fostering trust, accountability, and understanding among users.” The article ends by highlighting some possible future directions of these methods, such as the integration of AI with quantum computing frameworks.

Two of the mini‐reviews focus on the use of protein design approaches to develop antibody‐like molecules. In one of them, entitled “Structure‐based computational design of antibody mimetics: challenges and perspectives,” Chaves *et al*. [5] showcase several protein scaffolds that can be used as antibody mimetics, having several advantages over them, stemming from their smaller size and simpler molecular properties. An overview of different computational approaches that have been used to design these proteins is provided, which allows the reader to grasp how the recent changes in the protein design field have impacted the design pipelines. In the other mini‐review, entitled “Nanobody engineering: computational modelling and design for biomedical and therapeutic applications,” El Salamouni *et al*. [6] discuss how the structural features of nanobodies make them appropriate candidates for different applications, from diagnostics, to therapeutics and other biotechnological applications. This mini‐review provides a broad overview of different computational approaches that have been used to engineer these molecules, from standard MD simulations to enhanced sampling methods and more recently AI‐based approaches. This provides the reader with a clear perspective of how the field is evolving and of the possible future directions.

Another relevant topic in the field of Structural Bioinformatics is highlighted in the review entitled “Structural information in therapeutic peptides: Emerging applications in biomedicine” by Iglesias *et al*. [7], which showcases computational approaches that have been used to assist the discovery, design, and optimization of peptides toward specific applications. Key application examples, such as the discovery and development of antimicrobial and anticancer peptides, are discussed. An overview of the use of peptides for producing macromolecular assemblies (e.g., hydrogels and amyloid fibers) and as modifiers of protein aggregation is also provided. Focusing on these and other applications, the manuscript highlights the importance of taking structural and conformational features into account when developing peptides for therapeutic applications.

In the last manuscript, entitled “Viral entry mechanisms: the role of molecular simulation in unlocking a key step in viral infections,” by Valério *et al*. [8], we provide an overview of the main contributions provided by computational studies to our understanding of viral entry mechanisms, focusing on two main steps: viral attachment and viral membrane fusion. Viral entry, which is promoted by viral surface proteins, represents a crucial stage in viral infection and, thus, there is a tremendous interest in understanding the molecular mechanisms of this process. Computational methods, such as standard MD simulations, enhanced sampling methods, and other approaches, have been paramount for this quest by providing a detailed characterization of the interaction of viral fusion proteins with host receptors and by shedding light into the intricate details of membrane fusion. This mini‐review also discusses how this knowledge is already being applied in the development of inhibitors targeting viral fusion proteins.

We are certain this collection will constitute a useful roadmap for the readers of *FEBS Open Bio* who are trying to navigate through the recent developments in the field of Structural Computational Biology.

## Conflict of interest

The authors declare no conflict of interest.

## Author contributions

CMS and DL wrote the editorial.
